# Multiple intraosseous lipomatosis: a case report

**DOI:** 10.1186/1757-1626-2-7399

**Published:** 2009-05-15

**Authors:** Bhavya Rehani, Robert Wissman

**Affiliations:** Department of Radiology, University of Cincinnati Medical Center234 Goodman St., ML 0761, Cincinnati, Ohio 45267USA

## Abstract

**Introduction:**

Intraosseous lipoma is an uncommon entity that presents almost exclusively as a solitary lesion. Multiple intraosseous lipomas are exceedingly rare with only a few cases being reported in the literature.

**Case presentation:**

We present a case of 43-year-old African American female who presented with left leg and left wrist pain. The initial radiographs revealed well-defined radiolucent lesions in multiple bones involving the left wrist and the left lower limb. The magnetic resonance demonstrated multiple lesions, which showed high signal on the T1 and low signal on the fat suppressed T2 images. This favored the diagnosis of intraosseous lipomatosis that was confirmed by biopsy.

**Conclusion:**

Multiple intraosseous lipomatosis is an uncommon but important differential for multiple radiolucent lesions on the plain radiographs. This condition can lead to pathological fractures. Magnetic resonance imaging can aid in providing an accurate diagnosis. The awareness of this condition can help the clinician in guiding the correct diagnosis and management.

## Introduction

Multiple intraosseous lipomatosis is very rare and idiopathic entity. With few cases being reported in the literature [1-6] the etiology of this disease is still unexplained. It can lead to pathological fractures and thus the diagnosis of this condition is vital [3].

## Case Presentation

We present a case of an unemployed 43-year-old African American female who presented with bilateral leg pain. There was no history of trauma. Her past medical and surgical history was significant only for prior hysterectomy for fibroids and hormone replacement therapy. She denied smoking or drinking alcohol. The physical exam was significant for mild point tenderness on the medial aspect of her left knee. There was no joint effusion and no limitation in the range of movement.

**Figure 1.**
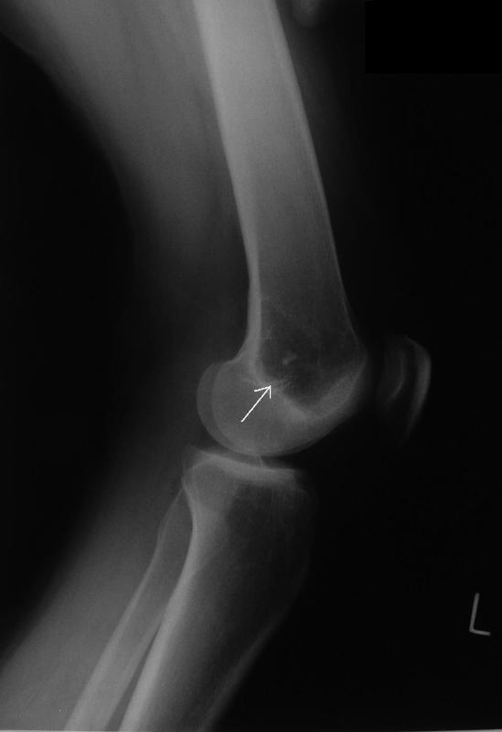


**Figure 2.**
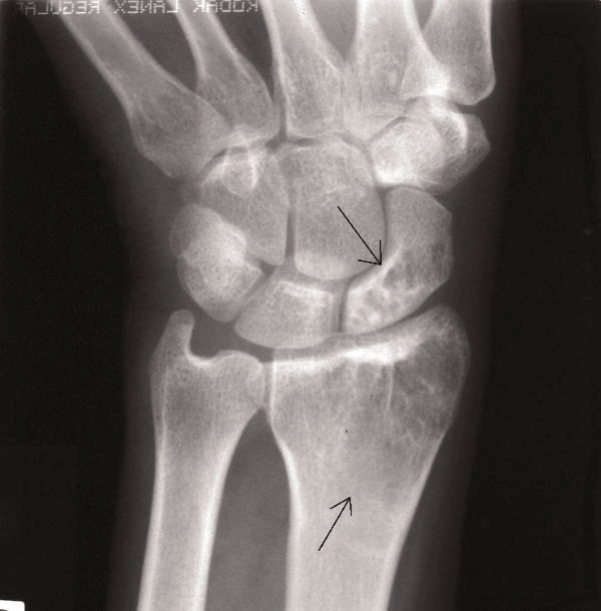


The plain film radiographs demonstrated well-defined lytic lesions of the left distal femur and the medial plateau (Figure 1). The plain film of the left wrist revealed multiple ill-defined radiolucent lesions in the distal radius and the scaphoid (Figure 2). The plain films of her right wrist and the right knee were normal.

Additional studies include complete blood count, liver, renal and thyroid function tests, serum lipid panel, serum electrophoresis, B+ T flow cytometry and bone marrow biopsy. The results of these tests were normal.

**Figure 3.**
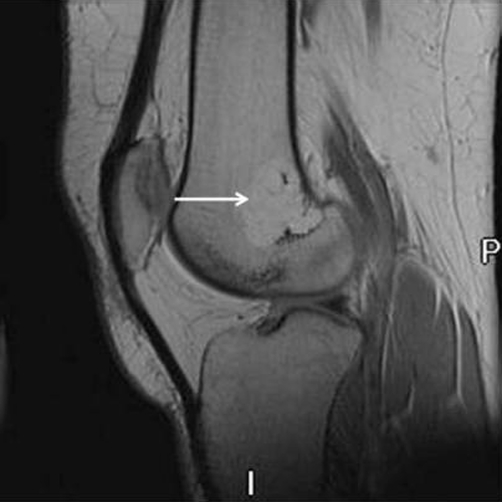


**Figure 4.**
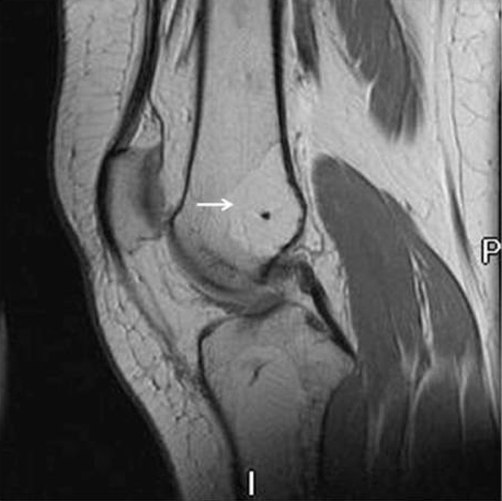


Magnetic resonance imaging (MRI) was subsequently done to evaluate these lesions further. MRI of the left knee and left wrist revealed regions of high T1 signal and low signal on the fat suppressed T2 images (Figures 3,4). These fat signal areas corresponded to the plain film lesions. There was no periosteal reaction or cortical break. The soft tissues were normal. In distal radius, there was an eccentric abnormality.

**Figure 5.**
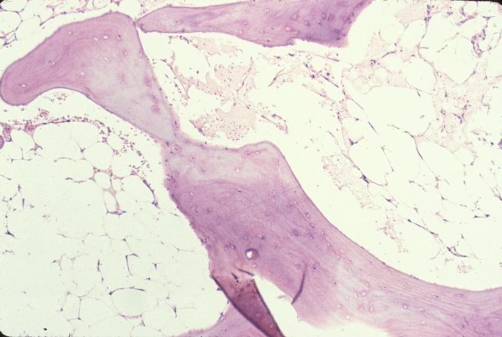


The biopsy of the medial tibial plateau lesion showed hyperplastic adipose tissue and confirmed the diagnosis of intraosseous lipomatosis. No malignant or atypical cells were visualized on the biopsy (Figure 5).

## Discussion

Intraosseous lipoma is a rare entity and was first described in 1880 [7,8]. Intraosseous lipomas are masses of lipocytes replacing the normal marrow that can undergo necrosis, calcification, cyst formation or bone resorption. Milgram has staged them into three types based on the occurrence of involutional changes. In stage 1, there is no secondary necrosis; in stage 2 there is partial necrosis and in stage 3 there is complete or near-complete necrosis. The increase in necrosis relates to increase in radiographic density and calcification. These can be misdiagnosed as bone infarcts in the later stages of involution [9]. The cause of these lesions is uncertain.

Intraosseous lipomas are almost always solitary lesions. Multiple bony involvements are unusual. There have been only a few case reports in the literature of multiple intraosseous lipomas [1-6]. Clinically, multiple intraosseous lipomas can present with pain or as an incidental findings on radiographs [9]. Intraosseus lipomas can be found in any part of the bone since fat is present in the marrow of all bones. However, the majority of these occur in the long bones and in the lower extremity [9,11]. Although the etiology is unknown, multiple intraosseous lipomas have been reported in the patients with hyperlipoproteinemia, macrodystrophia lipomatosa and as a congenital variant following autosomal pattern of inheritance [1,2,6]. These lesions can progress to pathological fractures [3]. In our patient, both the upper and lower limb involvement was seen and there was no coexisting condition that could have predisposed to this condition.

Imaging plays a vital role in the diagnosis of intraosseous lipomas. A correct radiological diagnosis can often be regarded as an endpoint to the investigation and biopsy or surgery can be avoided in the majority of cases [7]. On radiograph, these present as radiolucent lesions that progressively become radiodense with calcification and necrosis. The differential diagnosis includes the simple bone cysts, bone infarcts, fibrous dysplasia, aneurismal bone cysts, chondroid and myxoid tumors. CT demonstrates low attenuation of fat and surrounding thickened bony trabecula [11]. Bone scan demonstrates no to moderate positivity [12]. MRI contributes useful information for the correct diagnosis as the lesions follow fat signal. The lesions demonstrate increased signal on the T1 and decreased signal on the T2- weighted images [10,11]. The thin low signal rim on the T1 and T2- weighted images represent areas of reactive sclerosis [14]. There is lack of signal from the lesion on short-tau inversion recovery (STIR) sequence [11]. The central areas of low signal on the T1 and T2- weighted images represent areas of calcification in the later stages [14].

The management involves curette and graft of the symptomatic and progressive lesions [3,12]. Follow up can be helpful as these often progress to pathological fractures.

In summary, the knowledge of this unusual entity can help the clinician in explaining the patient's pain and aid in counseling the patient about the diagnosis, future risk of pathological fractures and the various treatment options.

## List of abbreviations

CT: Computer Tomography
MRI: Magnetic resonance imaging

## References

[bib-001] Freiberg RA, Air GW, Glueck CJ, Ishikawa T, Abrams NR (1974). Multiple intraosseous lipomas with type-IV hyperlipoproteinemia. A case report. J Bone Joint Surg Am.

[bib-002] Döhler R, Poser HL, Harms D, Wiedemann HR (1982). Systemic lipomatosis of bone. A case report. J Bone Joint Surg Br.

[bib-003] Szendroi M, Karlinger K, Gonda A (1991). Intraosseous lipomatosis. A case report. J Bone Joint Surg Br.

[bib-004] Rosenblatt EM, Mollin J, Abdelwahab IF (1990). Bilateral calcaneal intraosseous lipomas: a case report. Mt Sinai J Med.

[bib-005] Pande KC, Ceccherini AF, Webb JK, Preston BJ (1998). Intraosseous lipomata of adjacent vertebral bodies. Eur Spine J.

[bib-006] Whyte MP, Eddy MC, Podgornik MN, McAlister WH (1999). Polycystic bone disease: A new, autosomal dominant disorder. J Bone Miner Res.

[bib-007] Afip, Campbell RS, Grainger AJ, Mangham DC, Beggs I, Teh J, Davies AM (2003). Intraosseous lipoma: report of 35 new cases and a review of the literature. Skeletal Radiol.

[bib-008] Reig-Boix V, Guinot-Tormo J, Risent-Martinez F, Aparisi-Rodriguez F, Ferrer-Jimenez R (1987). Computed tomography of intraosseus lipoma of os calcis. Clin Orthop.

[bib-009] Milgram JW (1988). Intraosseus lipomas: Radiologic and Pathologic manifestations. Radiology.

[bib-010] Richardson AA, Erdmann BB, Beier-Hanratty S, Lautz D, Jacobs PM, Julsrud ME, Ringstrom JB (1995). Magnetic resonance imagery of a calcaneal lipoma. J Am Podiatr Med Assoc.

[bib-011] Levin MF, Vellet AD, Munk PL, McLean CA (1996). Intraosseous lipoma of the distal femur: MRI appearance. Skeletal Radiol.

[bib-012] Murphey MD, Carroll JF, Flemming DJ, Pope TL, Gannon FH, Kransdorf MJ (2004). From the archives of the AFIP: benign musculoskeletal lipomatous lesions. Radiographics.

[bib-013] Mena E, Estorch M, Camacho V, Fuertes J, Tembl A, Hernández MA, Flotats A, Carrió I (2004). Intraosseous lipomatosis: positivity in bone scintigraphy with 99mTc-HDP]. Rev Esp Med Nucl.

[bib-014] Propeck T, Bullard MA, Lin J, Doi K, Martel W (2000). Radiologic -Pathologic correlation of intraosseus lipomas. AJR Am J Roentgenol.

